# Intraoperative blood loss during different stages of scoliosis surgery: A prospective study

**DOI:** 10.1186/1748-7161-5-16

**Published:** 2010-08-07

**Authors:** Hitesh N Modi, Seung-Woo Suh, Jae-Young Hong, Sang-Heon Song, Jae-Hyuk Yang

**Affiliations:** 1 Scoliosis Research Institute, Department of Orthopedics, Korea University Guro Hospital, Seoul, Korea

## Abstract

**Background:**

There are a number of reasons for intraoperative blood loss during scoliosis surgery based on the type of approach, type of disease, osteopenia, and patient blood profile. However, no studies have investigated bleeding patterns according to the stage of the operation. The objective of this prospective study was to identify intraoperative bleeding patterns in different stages of scoliosis surgery.

**Methods:**

We prospectively analyzed the estimated blood loss (EBL) and operation time over four stages of scoliosis surgery in 44 patients. The patients were divided into three groups: adolescent idiopathic (group 1), spastic neuromuscular (group 2) and paralytic neuromuscular (group 3). The per-level EBL and operation times of the groups were compared on a stage-by-stage basis. The bone marrow density (BMD) of each patient was also obtained, and the relationship between per-level EBL and BMD was compared using regression analysis.

**Results:**

Per-level operation time was similar across all groups during surgical stage (p > 0.05). Per-level EBL was also similar during the dissection and bone-grafting states (p > 0.05). However, during the screw insertion stage, the per-level EBL was significantly higher in groups 2 and 3 compared to group 1 (p < 0.05). In the correction stage, per-level EBL was highest in group 3 (followed in order by groups 2 and 1) (p < 0.05). Preoperative BMD indicated that group 3 had the lowest bone quality, followed by groups 2 and 1 (in order), but the preoperative blood indices were similar in all groups. The differences in bleeding patterns in the screw insertion and correction stages were attributed to the poor bone quality of groups 2 and 3. Group 3 had the lowest bone quality, which caused loosening of the bone-screw interface during the correction stage and led to more bleeding. Patients with a T-score less than -2.5 showed a risk for high per-level EBL that was nine times higher than those with scores greater than -2.5 (p = 0.003).

**Conclusions:**

We investigated the blood loss patterns during different stages of scoliosis surgery. Patients with poor BMD showed a risk of blood loss nine times higher than those with good BMD.

## Background

Underlying disorders play major roles in determining blood loss during surgical procedures. In particular, patients with neuromuscular diseases are thought to be at risk for increased blood loss during scoliosis surgery, but most reports addressing this question are anecdotal [1-4]. The most common reasons postulated for observed increases in blood loss are related to the more extensive fusion required in patients with neuromuscular scoliosis. Studies that have examined specific neuromuscular disorders have found that blood loss increases in conditions in the following order: cerebral palsy (CP), spinal muscular atrophy (SMA), myelomeningocele, and Duchenne muscular dystrophy (DMD) [5]. It has also been found that posterior spinal fusion procedures tend cause more blood loss than anterior procedures, although this is due mostly to the greater number of vertebral levels fused in posterior approaches [5,6].

Posterior surgeries are becoming more common in scoliosis correction, especially since the introduction of third generation instruments. Some authors believe that even severe scoliosis can be corrected with a posterior-only approach using pedicle screws that provide strong purchase and correction forces [7-9]. Severe scoliosis can be corrected along with various posterior osteotomy procedures without anterior procedures, thus avoiding the risk of postoperative pulmonary compromise, which is especially relevant in cases of neuromuscular scoliosis. There is also variation in blood loss according to different types of scoliosis [9-13]. Some risk factors for intraoperative blood loss have been identified in patients with neuromuscular scoliosis. Edler et al. [14] note that neuromuscular patients have an almost seven times higher risk of developing complications after losing > 50% of their estimated total blood volume during scoliosis surgery. Osteopenic bones, decreased coagulation factor reserves [4], changes in the mitochondrial structure of vascular smooth muscle [15,16], and increased fibrinolytic activity [17] may affect hemostasis and lead to increased blood loss without frank coagulopathy.

A few studies have compared blood loss during scoliosis surgery in patients with neuromuscular disease and those without [5]. However, no studies have measured intraoperative blood loss during the different stages of scoliosis surgery. The aim of this study was to calculate the blood loss per level of fixation for different pathologies. We also assessed the relationship between blood loss patterns and preoperative bone quality.

## Materials and methods

We conducted a prospective analytical study to measure intraoperative blood loss during different stages of surgery in 44 patients who underwent operations between 2007 and 2008 performed by the senior surgeon (SWS) at our institute. The average age of the patients was 17.6 ± 7.3 years at the time of operation. There were 14 patients with AIS (group 1), 15 with spastic neuromuscular scoliosis (CP, group 2), and 15 with paralytic neuromuscular scoliosis (13 DMD, 2 SMA, group 3), respectively. After obtaining written informed consent, bone mineral density (BMD) was measured for each patient using a dual energy X-ray absorptiometry (DEXA) scan. Patients with pathologies other than those listed were excluded from the study. The average ages by group were 16.5, 21.8 and 14.4 years in groups 1, 2 and 3, respectively. All operations were performed using a single stage, posterior-only approach and pedicle screw fixation. Pedicle screws were inserted bilaterally at all levels. The correction maneuver for scoliosis was performed using the rod derotation technique with or without *in situ *bending. No additional anterior procedures or posterior osteotomies were performed. We did not include patients who required thoracoplasty for a rib hump. We divided the surgical procedure into four stages: stage 1-dissection; stage 2-screw insertion; stage 3-rod assembly followed by a correction maneuver with rod derotation and/or *in situ *bending; and stage 4-bone graft bed preparation followed by bone grafting and wound closure.

The hospital and clinical charts of all patients were reviewed, and information including indication of surgery, age, weight, preoperative medical conditions, blood bank consultations, perioperative records, laboratory information, repeated intraoperative haematocrit, platelet counts, and arterial blood gas level was collected. Fluid management data included records of intraoperative and postoperative crystalloids and colloids as well as the timing of autologous, directed donor or homologous blood. Autologous blood transfusions were performed postoperatively. Intraoperative homologous transfusions were completed by anesthetists. The estimated blood loss (EBL) was based on the amount of blood in the suction container (accounting for irrigation used on the surgical field) and the difference in the weights of dry and blood soaked sponges [5,14]. We measured the EBL for each stage and identified any differences across groups. We analyzed the per-level EBL and timing for each stage of the operation across the three groups using ANOVA and Tukey's *post hoc *analysis using SPSS (SPSS version 15, Chicago, IL, USA). Fixation levels were calculated by counting all levels from the proximal level of fixation to the distal level. Additionally, we examined preoperative blood indices and bone quality to identify any correlations between osteoporosis and per-level EBL.

## Results

**Figure 1:**
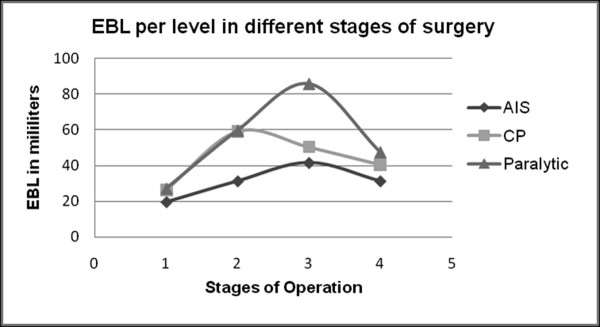
**Shows a diagrammatic chart of estimated blood loss (EBL) per level of fixation in the three patient groups**. On the x-axis, the numbers 1, 2, 3 and 4 correspond to the four stages of surgery: dissection (1), screw insertion (2), rod derotation (3) and bone grafting (4). The y-axis shows per-level EBL in milliliters.

Table 1 shows the diagnoses, preoperative and postoperative Cobb angles and fixation levels for all patients. The average preoperative Cobb angles were 75°, 85.4° and 68.5° for groups 1, 2 and 3 respectively. The average postoperative Cobb angles were 24.1° (70.9% correction), 23.9° (74.1% correction) and 18.9° (74.5% correction), and were similar for each group (p = 0.92, ANOVA). Additionally, the average preoperative flexibilities in groups 1, 2 and 3 were 41.3 ± 9.1%, 41 ± 11.3% and 46.8 ± 9.1%, respectively (Table 1). Comparing preoperative flexibilities by ANOVA showed no significant differences (p = 0.21). The average EBL for groups 1, 2 and 3 were 1523, 2667 and 3446 milliliters, respectively. The average operation times were 162.4, 215 and 217.4 minutes. There were an average of 12.1, 15 and 15.6 levels of fixation for groups 1, 2 and 3, respectively. Table 2 shows the per-level operation time and EBL for each patient. The per-level operation times for group 1 (13.6 min), group 2 (14.4 min) and group 3 (13.9 min) were not significantly different (p = 0.75, ANOVA and Tukey's post hoc test) (Table 2). However, per-level EBL was significantly different across the groups (group 1: 123.9 ml, group 2: 176.5 ml, and group 3: 220.7 ml) (p = 0.008, ANOVA). Table 3 shows per-level EBL according to stage. The dissection and bone grafting stages showed no significant differences across groups (dissection: p = 0.473, ANOVA and > 0.05 Tukey's *post hoc*, bone grafting: p = 0.276, ANOVA and > 0.05 Tukey's *post hoc*). However, we did observe significant differences in screw insertion (p = 0.028, ANOVA) and correction stages (p = 0.002, ANOVA); these factors were responsible for the overall differences we observed among all groups (Fig. 1). Analyzing the per-level EBL using Tukey's *post hoc *test during the screw insertion stage, we detected significant differences between groups 1 and 2 (p = 0.043) and groups 1 and 3 (p = 0.048), while the differences between groups 2 and 3 were not significant (p = 0.999). During the correction stage, we detected no significant differences between groups 1 and 2 (p = 0.748), while there were significant differences between groups 1 and 3 (p = 0.002) and groups 2 and 3 (p = 0.013). These results suggest that the paralytic group were characterized by increased per-level EBL compared to the CP and AIS groups, and that the CP group showed higher per-level EBL than the AIS group (but lower than the paralytic group). Our findings were further validated by rank-based Kruskal-Wallis test (Table 4).

**Table 1:** Mean age, preoperative and postoperative Cobb angle, number of fixation levels, operation time in minutes and EBL (estimated blood loss) in milliliters during the four surgical stages for each group.

Groups		Age	Cobb angle (°)			no of level	Time in min during different surgical stages					EBL in ml during different surgical stages				
						
			Pre op	Flexibility%	Post op		Dissection	Screw insertion	Rod derotation	Bone grafting	Total	D-EBL	S-EBL	R-EBL	B-EBL	Total
**Total**	**Mean**	17.6	76.3	43.1	22.3	14.3	60.3	78.8	35.7	24.3	199.1	350.8	749.7	884.7	584.3	2569.4
	**SD**	7.3	24.0	10.1	16.2	1.9	19.2	23.2	14.1	7.4	44.3	270.3	554.7	597.8	423.7	1455.9
**AIS (group 1)**	**Mean**	16.5	75.0	41.3	24.1	12.1	47.1	66.2	27.1	21.9	162.4	229.3	385.0	523.3	386.3	1523.9
	**SD**	9.1	25.3	9.1	19.4	1.4	13.5	16.8	7.8	5.3	26.3	164.9	171.3	379.8	265.5	669.0
**CP** **(group 2)**	**Mean**	21.9	85.5	41	23.9	15.0	66.9	79.7	43.5	24.9	215.0	391.1	906.9	762.3	607.6	2667.9
	**SD**	7.1	21.5	11.3	16.2	1.3	12.3	28.0	14.2	7.9	34.6	256.5	539.2	325.9	396.0	1015.6
**Paralytic (group 3)**	**Mean**	14.4	68.5	46.8	18.9	15.6	66.0	89.7	35.9	25.9	217.5	423.9	932.7	1344.3	745.7	3446.6
	**SD**	2.4	23.6	9.1	13.4	0.7	23.5	18.1	14.6	8.3	47.1	333.4	656.9	696.2	511.3	1770.8

Abbreviations: AIS: Adolescent idiopathic scoliosis; CP: cerebral palsy; D-EBL: Estimated Blood Loss in dissection stage (stage 1); S-EBL: Estimated Blood Loss during screw insertion stage (stage 2); R-EBL: Estimated Blood Loss during correction stage with rod derotation and in situ bending procedure (stage 3); B-EBL: Estimated Blood Loss during bone grafting-fusion and closure stage (stage 4).

**Table 2:** Per-fixation level surgical stage lengths for the three groups.

		Dissection			Screw insertion			Correction			Bone grafting			Total		
		
Group	Levels	Time	p value		Time	p value		Time	p value		Time	p value		Time	p value	
**AIS**	12.1	4	**0.507§**	0.475*	5.6	**0.761§**	0.916*	2.3	**0.097§**	0.142*	1.8	**0.609§**	0.674*	13.6	**0.75§**	0.734*
**CP**	15	4.5		**0.841#**	5.3		**0.942#**	2.9		**0.998#**	1.6		**0.648#**	14.4		**0.955#**
**Paralytic**	15.6	4.2		**0.808α**	5.7		**0.741α**	2.3		**0.15α**	1.6		**0.999α**	13.9		**0.885α**

P value suggestive of § ANOVA test. The symbols *, # and α indicate statistical significance of Tuckey's post *hoc test *between: *-AIS and CP groups, #-AIS and Paralytic groups, and α-CP and Paralytic groups.

**Table 3:** Per-fixation level EBL (estimated blood loss) during the four surgical stages for the three groups.

		Dissection			Screw insertion			Correction			Bone grafting			Total		
		
Group	Levels	EBL	p value		EBL	p value		EBL	p value		EBL	p value		EBL	p value	
**AIS**	12.1	19.6	**0.473§**	0.579*	31.3	**0.028§**	0.043*	41.6	**0.002§**	0.748*	31.3	**0.276§**	0.64*	123.9	**0.008§**	0.018*
**CP**	15	26.2		**0.498#**	59.3		**0.048#**	50.5		**0.002#**	40.5		**0.245#**	176.5		**0.006#**
**Paralytic**	15.6	27.2		**0.99α**	59.8		**0.999α**	86		**0.013α**	47.7		**0.743α**	220.7		**0.29α**

P value suggestive of § ANOVA test. The symbols *, # and α indicate statistical significance of Tuckey's *post hoc *test between: *-AIS and CP groups, #-AIS and Paralytic groups, and α-CP and Paralytic groups.

**Table 4:** Comparison of per level operation lengths and per level EBL in each stage with a Kruskal-Wallis rank test.

	Per level stage lengths					Per level EBL for each stage				
	
	Dissection	Screw	Correction	Bone grafting	Total	Dissection	Screw	Correction	Bone grafting	Total
**Chi-square**	1.26	9.609	10.216	1.503	8.223	1.474	0.369	4.862	1.037	0.943
**df**	2	2	2	2	2	2	2	2	2	2
**p value**	0.533	0.008*	0.006*	0.472	0.016	0.479	0.831	0.088	0.595	0.624

Ranks 1, 2 and 3 were groups 1, 2 and 3, respectively. df is the degrees of freedom and a * indicates significant differences.

Preoperative blood characteristics (hemoglobin level, prothrombin time, clotting time, platelet count and APTT) and average BMD values are shown in Table 5. We observed no significant differences in hemoglobin level (p = 0.46, ANOVA), prothrombin time (p = 0.81, ANOVA), clotting time (p = 0.5, ANOVA), platelet count (p = 0.32, ANOVA) or APTT (p = 0.98, ANOVA). However, the average BMD values were significantly different across the groups (p = 0.0001, ANOVA), indicating differences in bone quality according to pathology. Group 3 had the lowest bone quality (T score -2.9), followed by group 2 (T score--2.1) and group 1 (T score -1.2). Comparing per-level EBL with the BMD T-score with a cutoff point of -2.5 (i.e., osteoporosis) using regression analysis, we detected a significant relationship between blood loss and low T-scores (less than -2.5, p = 0.003), with an odds ratio of 9.02. Our findings suggest that patients with osteoporosis (T-score less -2.5) have a risk of high blood loss that is nine times higher than that of patients without osteoporosis.

**Table 5:** Average preoperative blood indices and pixel values.

	CBC (Hb)		PT		CT		Platelets		APTT		BMD	
	
Group	gm%	p value	%	p value	min	p value	n/cumm	p value	sec	p value	T-Score	p value
**AIS**	14.19	**0.46§**	93.75	**0.81§**	1.3	**0.5§**	277500	**0.32§**	33.91	**0.98§**	-1.2	**0.0001§**
**CP**	13.64		92.16		1.23		244923		33.93		-2.1	
**Paralytic**	14.12		94.37		1.02		245181		33.57		-2.9	

BMD: Bone Marrow Density by DEXA scan

§ suggestive of ANOVA test.

## Discussion

Children with neuromuscular scoliosis suffer greater intraoperative blood loss than children with idiopathic scoliosis [9,18]. Blood loss is correlated with several factors, including the number of levels that are operated on. Therefore, per-level EBL is an important parameter for understanding differences between outcomes in idiopathic and neuromuscular scoliosis [5]. In present study, we evaluated EBL per level of fixation. Additionally, we examined operation time and EBL per-level of fixation to detect any differences in bleeding according to pathology. We compared EBL during different stages of surgery to examine differences in blood loss patterns between patient groups. To our knowledge, this is the first paper to study the patterns of EBL per level of fixation in patients with scoliosis with different pathologies.

EBL also dictates whether an anterior or posterior approach is used. Anterior procedures are suggested to lead to less blood loss than posterior approaches, primarily due to the fact that there are less levels of fixation required in posterior procedures [2,11,13,19]. During posterior procedures, average per-level EBL was reported to be 65-150 ml, 100-190 ml and 200-280 ml in adolescent idiopathic, spastic neuromuscular (CP) and paralytic neuromuscular (DMD, SMA) scoliosis patients, respectively [5]. In the present study, average per-level EBL was 123.9 ml, 176.5 ml and 220.7 ml in idiopathic, CP and paralytic scoliosis patients, respectively, similar to previous reports. We also calculated per-level EBL during the stages of surgery as defined above. We excluded cases that required additional anterior or posterior procedures or thoracoplasty. Our study was well-controlled and restricted to four distinct surgical stages, with the data restricted to those gathered only during procedures that used a posterior approach and pedicle screw fixation. Our results demonstrate that the length of each stage was nearly the same across all three groups, but that per-level EBL differed according to group for some stages (Fig. 1). During the dissection stage, per-level EBL was the same in all three groups (p = 0.473), while it increased during the screw insertion stage in groups 2 and 3 (59.3 ml in group 2 and 59.8 ml in group 3 versus 31.3 ml in group 1). This increase in EBL was primarily due to bone bleeding in CP and paralytic patients. We observed a sudden increase in bleeding while entering the pedicle that was due to poor bone quality rather than epidural vessel injury. The bleeding patterns were also different during the correction stage: group 3 showed the highest level of bleeding, followed by groups 2 and 1, respectively. These findings again support our evaluations of bone quality in the three groups (Table 5). We also noticed some loosening at the bone-screw interface during the correction stage because of osteopenia, especially in the paralytic neuromuscular scoliosis group. This resulted in increased bleeding in group 3 compared to groups 2 and 1, especially in DMD patients. However, we did not record the numbers of screws that had actually loosened in each patient, which may mean that we omitted important information, although retrospective evaluation of loosened screws would not be accurate when the intention is to correlate numbers of screws with intraoperative EBL. Once the fixation and correction procedures were completed, the bleeding patterns observed during the final fusion stage were not significantly different across groups (p = 0.276). We suggest that the increased bleeding in groups 2 and 3 during the bone grafting stage was due to continuous oozing from the pedicle and decortication sites. Several factors may be responsible for the increased bleeding seen in the neuromuscular scoliosis patients, including subclinical hemostatic or coagulation abnormalities [17,20] or qualitative platelet disorder [9,21]. In combination, these factors could lead to both increased intrinsic blood losses and decreased clotting ability [14]. Brenn et al. [22] compared 17 cerebral palsy patients with 17 idiopathic scoliosis patients, and found that children with cerebral palsy develop significant alterations in parameters, especially after losing 15% of their blood volume, that may be related to increased blood loss. However, we observed increased blood loss in cerebral palsy patients during the screw insertion stage. During this stage, EBL did not exceed 15% of the total blood volume observed during the dissection stage. Moreover, the screw insertion and correction stages had higher EBL than the bone-grafting stage. This observation does not support the theory, because we expect maximum blood loss during the bone-grafting stage. Additionally the preoperative blood indices showed no significant differences across groups (Table 5). Group 2 included seven patients with a history of anticonvulsant medications and six without. We compared per-level blood loss across these groups and found no significant differences (p = 0.26, unpaired t-test) in per-level EBL. The preoperative stiffness of the curve can also affect intraoperative EBL, but, once again, we found no significant differences in preoperative flexibility across the three groups. Soft tissue conditions may also affect intraoperative EBL, but we controlled bleeding from the muscles and soft tissues during operations. We agree that soft tissue conditions may differ across AIS, CP and DMD patients; however, diligent control of bleeding from soft tissues did not make a major difference. Additionally, differences in EBL during different stages of the operation cannot be explained by only soft tissue conditions.

We also prospectively measured BMD for each patient, which is an important indication of bone quality. AIS patients showed the best bone quality, resulting in the least EBL per level. We agree that other factors, such as an altered bleeding profile during surgery, are also important and may result in greater blood loss in CP and DMD patients; however, when we compare both groups, the poorer bone quality observed in DMD patients compared to CP patients resulted in sudden increases in per-level EBL during the correction procedures.

We believe that this is the first study to assess per-level EBL during different stages of scoliosis surgery and to compare the results across three different pathologies. We found that there is an almost nine times higher risk of increased blood loss when a patient's T-score is less than -2.5. This finding underscores the possible role of bone quality in the outcomes of scoliosis surgery. Other factors, such as increased abdominal pressure with positioning or a curve correction procedure, some form of hypertensive anesthesia, blood transfusion at pre-determined levels of hemoglobin, early replacement of platelets and fibrinogen, and surgical techniques stressing rigorous mechanical wound hemostasis [23-26], may help control intraoperative bleeding during surgery. However, we ruled out the role of blood parameters, as they were similar in all three groups. Therefore, we believe that improving bone quality before surgery may decrease intraoperative blood loss and prevent further complications in scoliosis. However, further prospective studies are necessary to test this hypothesis.

## Conclusions

We studied the patterns of intraoperative blood loss during scoliosis correction surgery according to surgical stage and underlying pathology. Blood loss varies according to surgical stage and is related to degree of osteoporosis in individual patients. Patterns of blood loss may be explained by differences in bone quality, as well as other factors. Additional studies that examine these issues in more depth are warranted.

## Competing interests

The authors declare that they have no competing interests. Each author certifies that s/he has no commercial relationships (e.g., consultancies, stock ownership, equity interests, patent/licensing arrangements, etc.) that might pose a conflict of interest in connection with this article.

## Authors' contributions

HNM contributed to the conception and design of the experiment, data acquisition, analysis, and interpretation, and drafting and revising the manuscript. SWS contributed to the experimental conception and design, drafting of the manuscript, and gave final manuscript approval. SHS contributed to data acquisition and drafting of the manuscript. JHY contributed to data acquisition, critical revisions and final approval of the manuscript. JYH contributed to data acquisition, analysis, and interpretation.

All authors have read and approved the final manuscript.
